# A Bayesian approach to pilot-pivotal trials for bioequivalence assessment

**DOI:** 10.1186/s12874-023-02120-2

**Published:** 2023-12-19

**Authors:** Duo Lv, Michael J. Grayling, Xinyue Zhang, Qingwei Zhao, Haiyan Zheng

**Affiliations:** 1 Department of Clinical Pharmacy, The First Affiliated Hospital, Zhejiang University School of Medicine, Hangzhou, China; 2 Zhejiang Provincial Key Laboratory for Drug Evaluation and Clinical Research, Hangzhou, China; 3 Population Health Sciences Institute, Newcastle University, Newcastle, UK; 4 Department of Mathematical Sciences, University of Bath, Bath, UK

**Keywords:** Bioequivalence, Crossover design, Pilot data, Robustness

## Abstract

**Background:**

To demonstrate bioequivalence between two drug formulations, a pilot trial is often conducted prior to a pivotal trial to assess feasibility and gain preliminary information about the treatment effect. Due to the limited sample size, it is not recommended to perform significance tests at the conventional 5% level using pilot data to determine if a pivotal trial should take place. Whilst some authors suggest to relax the significance level, a Bayesian framework provides an alternative for informing the decision-making. Moreover, a Bayesian approach also readily permits possible incorporation of pilot data in priors for the parameters that underpin the pivotal trial.

**Methods:**

We consider two-sequence, two-period crossover designs that compare test (*T*) and reference (*R*) treatments. We propose a robust Bayesian hierarchical model, embedded with a scaling factor, to elicit a Go/No-Go decision using predictive probabilities. Following a Go decision, the final analysis to formally establish bioequivalence can leverage both the pilot and pivotal trial data jointly. A simulation study is performed to evaluate trial operating characteristics.

**Results:**

Compared with conventional procedures, our proposed method improves the decision-making to correctly allocate a Go decision in scenarios of bioequivalence. By choosing an appropriate threshold, the probability of correctly (incorrectly) making a No-Go (Go) decision can be ensured at a desired target level. Using both pilot and pivotal trial data in the final analysis can result in a higher chance of declaring bioequivalence. The false positive rate can be maintained in situations when *T* and *R* are not bioequivalent.

**Conclusions:**

The proposed methodology is novel and effective in different stages of bioequivalence assessment. It can greatly enhance the decision-making process in bioequivalence trials, particularly in situations with a small sample size.

**Supplementary Information:**

The online version contains supplementary material available at 10.1186/s12874-023-02120-2.

## Introduction

Bioequivalence studies aim to demonstrate if two pharmaceutical products, or two formulations of the same drug, provide similar levels of therapeutic activity [1]. Typically characterized by pharmacokinetic parameters, e.g., area under the plasma concentration-time curve (AUC) or maximum concentration (C$_\text {max}$), bioequivalence rests on the extent to which the active ingredient’s availability at the site of drug action and the absorption rate are equivalent. One main application of bioequivalence studies is in developing generic version(s) of a company’s brand-name drug that has gone through large-scale confirmatory trials. The generic substitution is expected to be commercially viable, because it does not have to repeat the complete and costly drug development process.

Regulatory agencies have now established guidelines [2, 3] to evaluate so-called average bioequivalence, population bioequivalence, and individual bioequivalence [4, 5]. This paper will concentrate on average bioequivalence, for which two-treatment, two-period, two-sequence ($2 \times 2$) crossover designs are a common approach to study design. The two one-sided test (TOST) procedure [6] has been widely applied for the analysis of such studies, owing to its simplicity. Specifically, the TOST features two composite null hypotheses (each set at level $\alpha$) for testing if the observed trial data are more extreme than two equivalence bounds, $\Delta _{L}$ and $\Delta _{U}$, respectively. When both null hypotheses are rejected, an overall significance level of $\alpha$ for the bioequivalence test can be claimed. Alternatively, one may compute a conventional $(1 - 2\alpha ) \times 100\%$ confidence interval for the geometric mean ratio of the AUC and/or C$_\text {max}$ of the test drug over its predecessor drug (i.e., a reference or registered drug), and see if it falls within a predefined bioequivalence range, i.e., $\Delta _{L}$ and $\Delta _{U}$ [7].

Bioequivalence pivotal trials typically entail a large enough sample size to ensure sufficient statistical power which allows for the detection of differences between the test and reference drugs. The FDA guidance outlines that the optimal sample size for a standard bioequivalence pivotal trial ranges from 24 to 36 subjects, with a minimum of 12 subjects required for each group [8]. For good practice, small-scale pilot studies are often conducted in clinical research to examine feasibility and gather information about the treatment effect before carrying out a full-scale pivotal trial [9]. This thinking also applies to bioequivalence assessment. That is, a pilot bioequivalence trial would often be run in a small number of volunteers (usually 8-12 subjects [10]) to improve, e.g., the blood sampling schedule, as well as to understand the pharmacokinetic profiles, intra-subject variability, etc. Potential interest in the preliminary evidence on bioequivalence, however, does not mean performing the TOST procedure at a conventional 5$\%$ significance level is appropriate, as is for pivotal bioequivalence trials. Some research has investigated the type I error rate control [11, 12] or estimation of the intra-subject variability [13] to determine the optimal sample size for bioequivalence assessment, under two-stage $2 \times 2$ crossover designs involving an internal pilot. Pan et al. [14] proposed a Pilot Acceptance Range (PAR) method to preliminarily establish bioequivalence for subsequent confirmation in a pivotal trial. In particular, the PAR method expands the specific range, i.e., ($\Delta _{L}$, $\Delta _{U}$) which is typically (80$\%$, 125$\%$), to a wider interval by accounting for the intra-subject variability as estimated from the pilot trial data. When two formulations are bioequivalent, the PAR method has a higher chance of recommending the conduct of a pivotal bioequivalence trial than the TOST procedure or the confidence interval approach.

Whilst a large majority of available methods for bioequivalence assessment are in the frequentist paradigm, several Bayesian analysis strategies have been proposed. These include Grieve (1994) [15], Ghosh and Khattree (2003) [16], Ghosh and Gonen (2008) [17], and Schuirmann et al. (2019) [18], wherein the use of vague priors is commonplace. In addition, Bayesian inference may be preferred over frequentist methods for crossover trials when advanced sampling techniques are thought as useful to obtain the joint distribution of parameters. In recent years, other enhancements of Bayesian applications in the bioequivalence field have been published. For example, de Souza et al. [19] developed a Bayesian methodology for bioequivalence trials in which a normality assumption on the data is not a prerequisite. Advantages of using a heavily-tailed distribution were further illustrated from a Bayesian perspective in the interest of handling outliers [20]. As far as we are aware, though, statistical literature in this field has been written vastly for analysing pivotal bioequivalence trials, whereas scant attention has been paid to decision-making using the pilot data.

In this paper, we propose a novel Bayesian decision framework involving a robust Bayesian mixed-effects model to inform whether to conduct a pivotal bioequivalence trial following a pilot evaluation. We assume both the pilot and pivotal trials are carried out under a $2 \times 2$ crossover design. If continuing the assessment with a pivotal trial, the proposed Bayesian model is also capable of using all available data to establish average bioequivalence in a final analysis. The operating characteristics of the proposed methodology are evaluated in a comprehensive simulation study, as elaborated in the Results section. As motivation for our work, the Methods section in the following also contains a real pilot trial that aimed to preliminarily establish bioequivalence of two Pantoprazole tablets, a proton pump inhibitor for reducing gastric acid secretion.

## Methods

### Motivating example

A total of 12 healthy volunteers were enrolled to a pilot trial, using a $2 \times 2$ crossover design, to preliminarily assess the bioequivalence of two formulations of Pantoprazole tablet 40 mg in China. The venous blood was collected at 16 time points in each period, namely, within 10 min before drug administration (0 h), 20 min, 40 min, 1.0, 1.5, 2.0, 2.5, 3.0, 3.5, 4, 5, 6, 8, 10, 12 and 16 h after treatment. In this study, AUC$_\text {0-t}$ (the area under the concentration time curve from time 0 to the last time observed) was used to represent AUC. The pharmacokinetic parameters AUC$_\text {0-t}$ and C$_\text {max}$ were calculated by Phoenix WinNonlin software (version 7.0, Certara, Inc., Princeton, NJ, USA). An ANOVA test was performed for each pharmacokinetic parameter based on the Pantoprazole data with the key results listed in Supplement Table S1.

Due to data confidentiality, we simulated a pharmacokinetic dataset based on some summarised study characteristics instead of sharing the original data. The simulated pilot trial data was analysed using the TOST procedure with a $90\%$ confidence interval (CI). The geometric mean ratios were 116.78$\%$ (96.59$\%$ - 141.20%) for C$_\text {max}$ and 105.13% (93.10% - 118.72%) for AUC. Comparing the $90\%$ CIs, with the conventional bioequivalence limits (i.e., 80$\%$ - 125$\%$), inference using C$_\text {max}$ does not suggest preliminary bioequivalence in the pilot trial. The investigation would then be halted and no full-scale pivotal trial was to take place for formal bioequivalence assessment. By inspecting the subject-level data, we found that plasma concentrations of two subjects differed substantially from the others. The conclusion would change if the outliers are removed to mitigate the sampling error. With such an approach, the 90$\%$ CI method suffers even more from the small sample size of the pilot trial.

The following two questions are raised:

i. How should a $2 \times 2$ crossover pilot trial be analysed to inform a Go/No-Go decision for a pivotal bioequivalence trial?
ii. On the completion of the pivotal bioequivalence trial, is there scope for using pilot data, particularly in situations of data consistency?

### A robust Bayesian mixed-effects model for pilot-pivotal trials

**Fig. 1:**
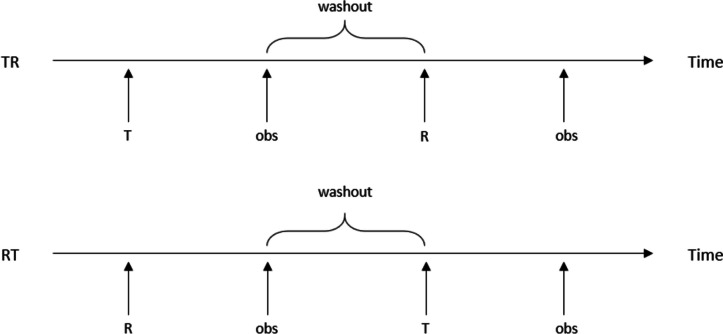
A schematic of 2$\times$2 crossover trials to establish average bioequivalence between two drugs, labelled *T* and *R*

Consider a $2 \times 2$ crossover design for comparing a test drug (labelled *T*) with a reference drug (labelled *R*). Patients are randomised into two treatment sequence groups: the first sequence will administer *T* during the first period and *R* during the second period, while the second sequence will administer the same treatments in reverse order. Figure 1 is a schema of such $2 \times 2$ crossover trials. We assume a washout period is included, between the two treatment periods, that is sufficiently long and thorough so that there is no carryover effect across the periods.

We let $Y_{ijk}$ be the continuous outcome, i.e., the logarithmic AUC or C$_\text {max}$, measured from subject *i* in sequence *j* during the *k*th period, for $i = 1, \dots , m_j$, $j = 1, 2$, and $k = 1, 2$. We define dummy variables $P_j$ to indicate the period and $X_{i[jk]}$ the treatment specific to the *i*th subject. Precisely, $j \ne k$ specifies the administration of *R* whilst $j = k$ specifies that of *T* by the design. We further stipulate that $X_{i[jk]} = 0$ (for $j\ne k$) or 1 (for $j=k$); $P_j = 0$ (for period 1) or 1 (for period 2). The following linear mixed-effects model has been widely considered to fit the trial data:

1 $$\begin{aligned} Y_{ijk}&\sim N(\mu _{ijk}, \sigma _w^2)\nonumber \\ \mu _{ijk}&= \beta _{01} + \beta _{11} P_j + \theta _1 X_{i[jk]} + e_{ij}, \quad e_{ij} \sim N(0, \sigma _b^2), \end{aligned}$$

where $\beta _{01}$ is an intercept term (i.e., the mean response on receiving *R* in period 1), $\beta _{11}$ is a fixed period effect, and $\theta _1$ is a fixed direct treatment effect specific to the pilot trial. This model also accommodates within-subject random error and a between-subject random effect, denoted by $\sigma _w^2$ and $\sigma _b^2$ respectively.

We now focus on the pivotal trial. Likewise, we let $Y^{\star }_{ijk}$ denote the continuous outcome and $\mu _{ijk}^\star$ the mean for subject *i* in sequence *j* during the *k*th period, where $i = 1, \dots , n_j$, $j = 1, 2$, and $k = 1, 2$. Letting $P_j^\star$ and $X_{i[jk]}^\star$ denote the corresponding dummy variables, we fit the following data model

2 $$\begin{aligned} Y^{\star }_{ijk}&\sim N(\mu _{ijk}^\star , \xi _w^2)\nonumber \\ \mu _{ijk}^\star&= \beta _{02} + \beta _{12} P_j^\star + \theta _2 X_{i[j,k]}^\star + r_{ij}, \quad r_{ij} \sim N(0, \xi _b^2), \end{aligned}$$

where $\xi _w^2$ and $\xi _b^2$ are the respective within-subject and between-subject variances, and $\theta _2$ is a direct treatment effect specific to the pivotal trial.

We consider a meta-analytic framework [21] to accommodate both the pilot and pivotal data, by stipulating a Bayesian hierarchical random-effects models. A predictive distribution can thus be obtained for $\theta _2$ based on the pilot data, hereafter denoted by $\varvec{Y}_p$. To relate the study-specific treatment effects, a normal-normal hierarchical model assumes that $\theta _1$ and $\theta _2$ are exchangeable, i.e., random samples drawn from an underlying normal distribution with unknown mean $\lambda$ and variance $\tau ^2$. That is, $\theta _1, \theta _2 \mid \lambda , \tau ^2 \sim N(\lambda , \tau ^2)$. The unknown population variance $\tau ^2$ characterises the between-study heterogeneity. A small value of $\tau ^2$ suggests considerable data consistency, so pilot data can be used to a large extent to predict the bioequivalence in the pivotal trial. In light of possible data inconsistency, we follow Neuenschwander et al. [22] to consider a robust version of the normal-normal hierarchy for the parameter model

3 $$\begin{aligned} \begin{array}{rcl} \theta _1 \mid \lambda , \tau ^2 \sim N(\lambda , \tau ^2) &{}\text {with prior probability} &{} \; 1, \\ \theta _2 \mid \lambda , \tau ^2 \sim N(\lambda , \tau ^2) &{}\text {with prior probability} &{} \; w, \\ \theta _2 \sim N(m, s^2) &{}\text {with prior probability} &{} \; 1 - w. \end{array} \end{aligned}$$

Model (3) comprises a robust component such that $\theta _2$ could have its own $N(m, s^2)$ prior. The prior variance $s^2$ is often set to a large value, so that inference about $\theta _2$ can utilize the pivotal data alone in situations of non-exchangeability. These prior probabilities reflect the level of confidence in data consistency *a priori* across the pilot and pivotal trials. More detail will be given in the next subsection about the specification of *w* at different stages of the bioequivalence assessment. Our Bayesian model is completed by specifying the hyper-prior distributions:

4 $$\begin{aligned} \lambda \sim N(a, b^2), \qquad \tau ^2 \sim HN(c), \end{aligned}$$

where *HN*(*c*) denotes a half-normal distribution. That is, a $N(0, c^2)$ distribution truncated to positive real numbers only. In general, *a* and $b^2$, are chosen to make the hyper-prior weakly informative, while *c* chosen to capture the plausible degree of between-study variability in terms of the treatment effects.

This robust Bayesian hierarchical model features a pair of prior probabilities of exchangeability and non-exchangeability, and thus accounts for situations where $\theta _2$ is not exchangeable with $\theta _1$. Setting *w* to a value close to 1 would mean high level of confidence in the relevance of the pilot trial and the data consistency. This robust model can also well accommodate situations of data inconsistency: if drastically different from the treatment effect based on pilot data, $\theta _2$ can be estimated using the pivotal trial data alone under a weakly-informative prior, $N(m, s^2)$. A similar modelling strategy was adopted by Zheng et al. [23] to incorporate preclinical data from one or multiple animal species in the design and analysis of phase I dose-escalation trials and later extended for situations when the phase I trial involves potentially heterogeneous patient subpopulations [24].

Recall that the data models (1) and (2) include covariates, that is, the treatment period. We do not consider exchangeability for the coefficients of the period effects, since it is more plausible that these may differ considerably between the pilot and pivotal bioequivalence trials. However, this could be an option for further research or considered as plausible in a context wherein the investigator has sufficient assurance. Vague priors are specified for these coefficients, as well as for the intercept terms, and within- and between-subject variances in each data model:

5 $$\begin{aligned} \begin{array}{ll} \beta _{0\ell } \sim N(0, 1000), &{} \beta _{1\ell } \sim N(0, 1000), \qquad \text { for } \ell = 1, 2, \\ \sigma _w^2 \sim \text {Inv-Gamma}(0.001, 0.001), &{} \sigma _b^2 \sim \text {Inv-Gamma}(0.001, 0.001), \\ \xi _w^2 \sim \text {Inv-Gamma}(0.001, 0.001), &{} \xi _b^2 \sim \text {Inv-Gamma}(0.001, 0.001). \end{array} \end{aligned}$$

The established Bayesian hierarchical model leads to a predictive distribution for $\theta _2$, denoted by $p(\theta _2 \mid \varvec{Y}_p)$. It can be approximated by a mixture of two component distributions [21]. In what follows, we illustrate how this predictive distribution can be (i) used to yield a Go or No-Go decision for carrying out a pivotal bioequivalence trial, and (ii) updated by the pivotal trial data, denoted by $\varvec{Y}^\star _p$, to a robust posterior distribution for the formal bioequivalence assessment. For probabilistic inference, one can sample the predictive distribution, $p(\theta _2 \mid \varvec{Y}_p)$, as well as the posterior distribution, $p(\theta _2 \mid \varvec{Y}_p, \varvec{Y}^\star _p)$, using Markov Chain Monte Carlo methods.

### Bayesian decision criteria to establish bioequivalence in two steps

In line with the convention, we stipulate that a Go decision would be allocated on the basis of pilot data if the following criteria is met

6 $$\begin{aligned} \mathbb {P}(\log (\gamma ^{-1}\cdot \Delta _L)\le \theta _2 \le \log (\gamma \cdot \Delta _U) \mid \varvec{Y}_p) > \eta , \end{aligned}$$

and a No-Go decision otherwise. Here, $\gamma$ is a scaling factor that should be set to a value not smaller than 1, and $\eta$ is a probability threshold. With $\gamma = 1$, this decision criterion becomes a Bayesian analogue to the conventional method of assessing bioequivalence. By contrast, $\gamma > 1$ results in a wider interval than $(\Delta _L, \Delta _U)$ that is required by regulatory agencies, so the criterion is less stringent. The latter is particularly useful to establish preliminary bioequivalence based on pilot data alone.

The sample sizes for both the pilot and pivotal trials would typically be chosen before the conduct of either trial. More often than not, maximally 12 subjects are included in a pilot trial; whereas, the sample size for a pivotal trial is calculated formally to ensure a desired statistical power of the TOST procedure at a certain significance level. Such calculation relies on a plausible value for the within-subject variance, rather than the estimate of $\sigma _w^2$ based on the pilot data, unless the assumed value is found very unreasonable. Despite the potential utility of, and interest in, sample size (re-)calculation, we consider this to be beyond the scope of this paper. In what follows, we concentrate on defining the scaling factor, $\gamma$, given known sample sizes for the pilot and pivotal trials.

After the pilot data becomes available, a posterior for $\theta _1$ can be obtained. Let an interval $(a, a +\ell _1)$ be symmetric about the posterior mean of $\theta _1$. The half-width of the interval can be defined to retain a coverage probability of $(a, a +\ell _1)$ as $100(1-2\alpha )\%$; that is, $\ell _1/2 = z_\alpha \sigma _{\theta _1}$, where $z_\alpha$ is the upper $\alpha$th quantile of the standard normal distribution and $\sigma _{\theta _1}$ the posterior standard deviation of $\theta _1$. Its counterpart in the pivotal trial is denoted by $\ell _2/2 = z_\alpha \sigma _{\theta _2}$. The (half-)width of the credible interval varies with the sample size, that is, a larger sample size yields narrower width of the interval. Following the notation defined in the last subsection, we denote the number of subjects by $m_j$ for sequence $j = 1, 2,$ in the pilot trial and likewise by $n_j$ in the pivotal trial. Specifying the scaling factor as

7 $$\begin{aligned} \gamma = \exp \left[ z_{\alpha }\sigma _{\theta _1} \left( \sqrt{n_1^{-1} + n_2^{-1}} - \sqrt{m_1^{-1} + m_2^{-1}}\right) \right] , \end{aligned}$$

can potentially account for the possible sampling error arising from the typically small sample size of the pilot trial. For the convention, by which $m_j < n_j$ for $j = 1, 2$, this stipulation leads to $\gamma > 1$ and gives a relaxed condition for declaring preliminary bioequivalence. In the special case when the pilot trial has a sample size equivalent to that of the pivotal trial, $\gamma = 1$ such that the same standard for a formal bioequivalence assessment applies.

We would like to add one more note on the specification of the prior mixture weight, as it affects the estimation of $\theta _2$. A general recommendation would be setting *w* to a high value close to 1 for eliciting a Go/No-Go decision using the pilot data alone, yet to a low value such as 0.1 or 0.2 for the final analysis to formally establish the bioequivalence largely based on the pivotal trial data. This would then allow $\varvec{Y}^\star _p$ to dominate the posterior distribution. Numerical exploration of various choice for *w* will be performed in the subsequent section. Finally, average bioequivalence can then be established, using all available data on the completion of a pivotal trial, if

8 $$\begin{aligned} \mathbb {P}(\log (\Delta _L)\le \theta _2 \le \log (\Delta _U) \mid \varvec{Y}_p, \varvec{Y}^\star _p ) > \eta . \end{aligned}$$

## Results

### Simulation setup

In this section, we evaluate the operating characteristics of the proposed Bayesian hierarchical model in comparison with two alternative methods in the frequentist hypothesis testing framework.

Motivated by an analysis of the original Pantoprazole pilot trial (see Supplement Table S1 for the summary of results), we construct nine simulation scenarios in Supplement Table S2 that feature various geometric mean ratios (${\text {GMR}} = \exp \left( \mu _{\textrm{T}}\right) / \exp \left( \mu _{\textrm{R}}\right)$) for a wide range of direct treatment effects. Pilot data are generated according to these nine scenarios, assuming a total sample size of 12 throughout. In Scenarios 3 – 7 (with 0.80 $\le$ GMR $\le$ 1.25), the two formulations *T* and *R* are bioequivalent. Accordingly, it would thus be desirable to yield a Go decision for conducting the subsequent pivotal trial. By contrast, a No-Go decision would be expected in the other scenarios.

In addition, it is of interest to understand how the proposed methodology reacts to potential data (in)consistency across the pilot and pivotal trials. We performed additional simulations of pivotal trial data under five bioequivalent cases (labelled A-E, with GMR = 0.8, 0.9, 1.0, 1.1, 1.25) and four inequivalent cases (with GMR = 0.5, 0.7, 1.43, 2.0). All scenarios assumed a total sample size of 24. After a Go decision is allocated following the pilot trial, the pivotal trial will be undertaken. To formally establish bioequivalence using both pilot and pivotal trial data, we utilized the perfect bioequivalence scenario of the pilot trial, which had a GMR of 1.0. The power of the bioequivalence assessment was estimated in simulations where treatment effects were consistent between the pilot (GMR = 1.0) and pivotal trials (0.80 $\le$ GMR $\le$ 1.25). Conversely, the probability of incorrectly declaring bioequivalence was estimated in simulations with inconsistent treatment effects between the pilot (GMR = 1.0) and pivotal trials (GMR $< 0.80$ or $> 1.25$).

Per simulation scenario and per case of data (in)consistency, a total of 10,000 replicates are simulated for the pilot and pivotal trials.

### Simulation results

In this simulation study, the within-subject variance $\sigma _w^2$ is higher in C$_\text {max}$ (23.7%) than AUC (12.2%) , as Table S1 shows.

Focusing on the analysis of pilot data first. The computed probability of a correct Go can be regarded as a *sensitivity* measure, while the probability of correct a No-Go can be regarded as the *specificity* in the analogue of terms typically used when evaluating a diagnostic test. Table 1 shows the probability of making a Go decision towards a subsequent pivotal trial by the conventional CI method (for $\alpha = 0.05$, 0.1 and 0.2) and the PAR method. Details on these conventional approaches is given in Appendix I. Not surprisingly, the conventional CI method ($\alpha = 0.05$ and 0.1) gives overly conservative results for a No-Go decision in Scenarios 3-7, where preliminary bioequivalence should be suggested. By contrast, the PAR method yields a higher the probability of a Go by accounting for varying extents of the intra-subject variance. The advantage of the PAR method manifests, as compared with the CI method ($\alpha = 0.05$ and 0.1), especially when the pilot trial has a small sample size and/or the data within the trial has large variability. For example, it increases from 31.19% or 56.28% by the CI method (corresponding to $\alpha = 0.05$ or 0.1, respectively) but to 76.41% by the PAR method in Scenario 5 for C$_\text {max}$; whereas the increase appears more marginal for AUC, given the much smaller within-patient variability. The probability using the CI method ($\alpha$ = 0.2) is slightly higher compared to the PAR method, with the difference within 5%. In the evaluation of our proposed Bayesian method below, we report the simulation results for both C$_\text {max}$ and AUC to understand the operating characteristics dependence on the level of variability.

**Table 1:** Probability of making a Go decision (%) to implement a pivotal trial, based on different methods to establish average bioequivalence

Comparator	$\eta$	(*w*, 1 - *w*)	Sc. 1	Sc. 2	Sc. 3	Sc. 4	Sc. 5	Sc. 6	Sc. 7	Sc. 8	Sc. 9
			GMR = 0.5	GMR = 0.7	GMR = 0.8	GMR = 0.9	GMR = 1.0	GMR = 1.1	GMR = 1.25	GMR = 1.43	GMR = 2.0
CI method ($\alpha$ = 5%)											
C$_\text {max}$			0	0.24	4.23	20.35	31.19	21.74	4.31	0.19	0
AUC			0	0.01	4.45	51.68	88.20	56.90	5.20	0	0
CI method ($\alpha$ = 10%)											
C$_\text {max}$			0	0.58	9.11	38.61	56.28	41.00	9.81	0.55	0
AUC			0	0.03	9.33	67.84	95.52	72.91	9.94	0.05	0
CI method ($\alpha$ = 20%)											
C$_\text {max}$			0	1.71	19.56	59.70	79.67	62.38	20.35	1.67	0
AUC			0	0.14	19.37	82.84	98.87	86.42	20.03	0.15	0
PAR method											
C$_\text {max}$			0	1.22	16.69	55.44	76.41	58.89	17.90	1.29	0
AUC			0	0.10	16.53	80.18	98.56	84.10	17.28	0.11	0
Proposed method											
C$_\text {max}$	0.85	(0.9, 0.1)	0	1.99	21.60	62.75	81.90	64.61	21.22	1.84	0
AUC	0.85	(0.9, 0.1)	0	0.15	20.32	83.99	99.02	86.95	20.72	0.19	0
C$_\text {max}$	0.85	(1.0, 0)	0	5.33	37.07	78.48	92.88	80.91	36.83	4.83	0
AUC	0.85	(1.0, 0)	0	0.75	36.33	92.73	99.85	94.36	36.14	0.70	0
C$_\text {max}$	0.95	(1.0, 0)	0	1.53	18.52	58.14	78.13	59.66	18.41	1.32	0
AUC	0.95	(1.0, 0)	0	0.13	17.89	81.36	98.67	84.64	17.89	0.13	0

CI method: Confidence Interval Method; $\alpha$, significance level

PAR method: Pilot Acceptance Range method

Proposed method: the proposed robust Bayesian hierarchical model with a scaling factor $\gamma$

We implement the proposed method with two configurations of the probability threshold and the prior mixture weight, i.e., (i) $\eta = 0.85$, $w = 0.9$, and (ii) $\eta = 0.95$, $w = 1.0$. As Table 1 illustrates, our Bayesian method outperforms both the CI method and the PAR method. In Scenarios 4 - 6, the probability of a Go decision is 62.75%, 81.90%, and 64.61%, respectively (under the configuration of $\eta = 0.85$, $w = 0.9$). This becomes 78.48%, 92.88%, 80.91%, when $\eta = 0.85$ and $w = 1.0$. In comparison with the PAR method, which yields 55.44%, 76.41% and 58.89% in scenarios 4-6, and the CI method ($\alpha$ = 0.2), which yields 59.70%, 79.67% and 62.38% in the same scenarios. By contrast, the proposed method improves upon the decision making to correctly allocate a Go decision in scenarios of bioequivalence and not to allocate a Go otherwise. The probability of a Go decision decreases to 58.14%, 78.13%, 59.66%, when $\eta$ is raised to 0.95 alongside a full prior mixture weight, $w = 1.0$. The choice of $\eta$ will evidently impact the likelihood of making a Go decision. If $\eta$ is calibrated to a lower value whilst retaining all other parameters, the probability of a Go decision increases. Accordingly, the proposed method could have performed far superior to the conventional approaches with a lower threshold in this simulation study. It is nonetheless worth noting that the probability of an incorrect Go decision would be higher. The proposed method may yield high probability of bioequivalence for borderline values of 0.8 or 1.25, if not coupled with a stringent threshold, leading to an increased likelihood of conducting a pivotal study. In contrast, the CI method adheres to the $\alpha$ level.

**Fig. 2:**
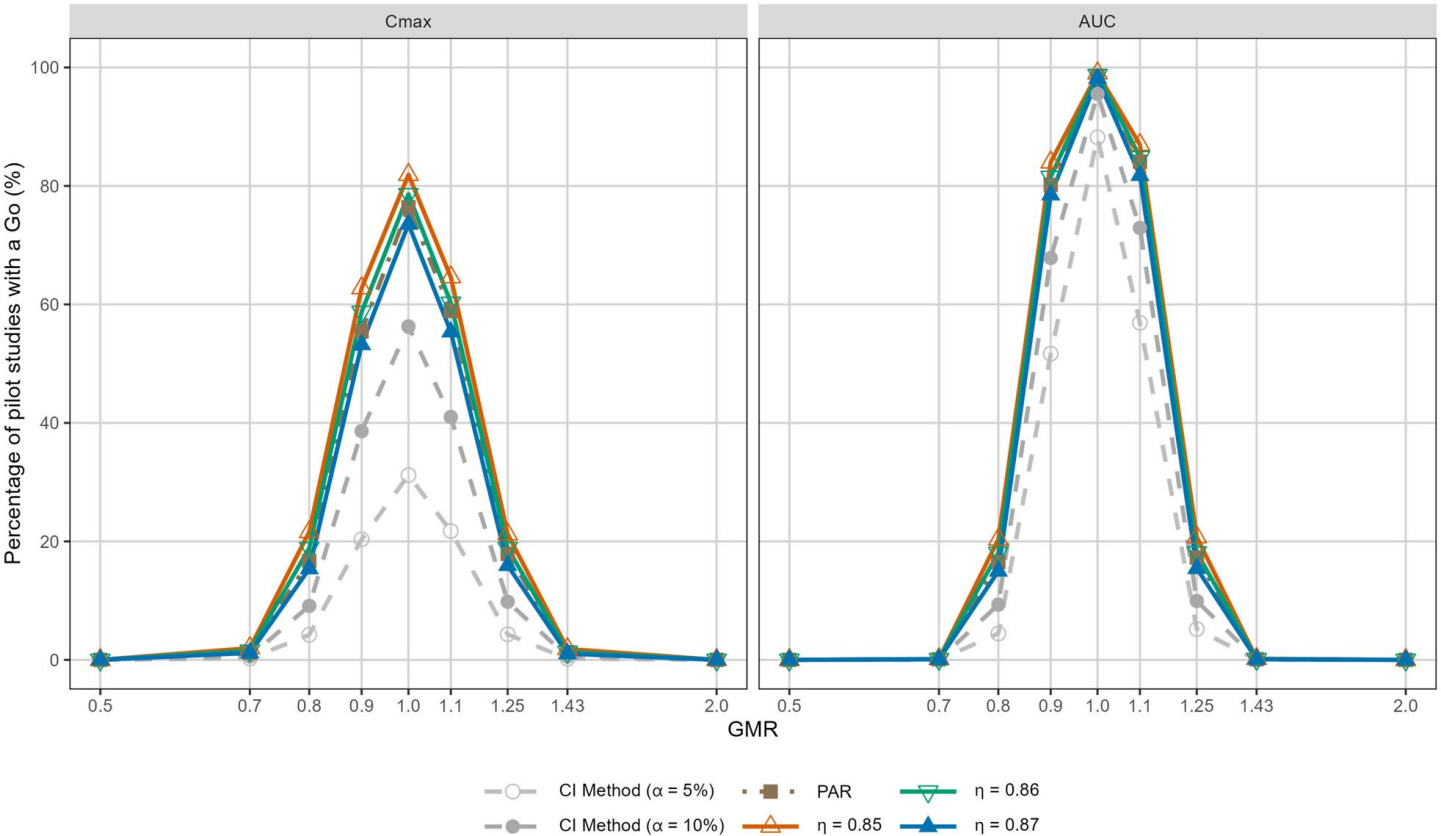
Probability of a Go decision (%) towards a pivotal trial yielded by the proposed method specifying the prior mixture weights, (*w*, 1 - *w*) = (0.9, 0.1), coupled with various choices of the threshold, $\eta$ = 0.85, 0.86, 0.87, respectively. Choosing a value below 0.85 for $\eta$ will produce higher chance of a Go decision under the same GMR scenario

In practice, we recommend investigators perform extensive simulations to understand how the operating characteristics would be modified by $\eta$ so as to choose an appropriate value. To illustrate how this could be achieved, we expand the simulation study to quantify the likelihood of Go/No-Go decisions under various configurations of $\eta$. Figure 2 visualises the probability of a Go decision towards a pivotal trial, setting $\eta = 0.85$, 0.86, 0.87, together with $w = 0.9$, for the simulated C$_\text {max}$ and AUC data. As one may observe, the lines cannot be disengaged in the inequivalent scenarios (i.e., when GMR = 0.5, 0.7, 1.43, 2.0). The differences eventually become observable, that is, the proposed Bayesian model surpasses the conventional methods in the bioequivalent scenarios (i.e., when GMR = 0.8, 0.9, 1.0, 1.1, 1.25). Here we have presented results by setting $\eta$ to a value ranging from 0.85 to 0.87. The PAR method has comparable performance to the proposed method with $\eta = 0.87$, whilst it becomes marginally inferior to our method when $\eta = 0.85$ and 0.86. Although not shown in Fig. 2, our method gains a higher chance of a correct Go decision if the value for $\eta$ is further decreased. The user is thus recommended to adopt our method with the decision threshold set to be 0.85 at maximum in this illustration. More simulations have been performed for configurations of $w = 0.8$ and $w = 1.0$, as shown in Figs. S1–S2, to find an appropriate decision threshold that ensures the benefit of our proposed method.

**Fig. 3:**
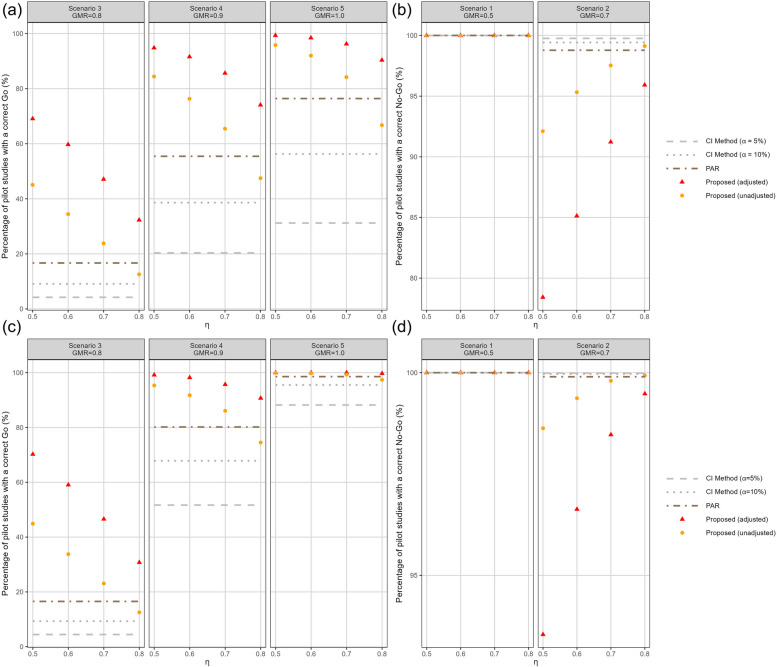
Probability of making correct Go or correct No-Go decision (%) towards a pivotal trial using different methods and decision frameworks to establish the average bioequivalence. The proposed method was implemented with (*w*, 1 - *w*) = (0.9, 0.1), with the ‘adjusted’ being the specification of $\gamma$ following Eq. (7) and the ‘unadjusted’ being $\gamma$ = 1. (a) and (b) using C$_\text {max}$ data. (c) and (d) using AUC data

Figure 3 summarises the percentages of correct Go or correct No-Go decisions for the simulated pilot studies under the configuration with $w = 0.9$. The proposed method (‘adjusted’) specifies $\gamma = 1$ following Equation (7) and the ‘unadjusted’ method specifies $\gamma > 1$. The correct Go percentage can be resembles the frequentist power, while the correct No-Go percentage related to the frequentist type I error rate (specifically, 1 - $\alpha$). For both metrics higher values are better. On the log-scale, the results of CI, PAR and the proposed Bayesian models are symmetric around 0 in bioequivalence assessments. Furthermore, the results of the GMR, i.e. 0.8 and 1.25, 0.7 and 1.43, 0.9 and 1.11, are congruent. To enhance the figures’ legibility, we solely showcase the faceted plots for the GMR $\le$ 1 in Figs. 3 and S3. In Scenarios 3 - 7, the bioequivalent scenarios, the percentage of a correct Go decision declines as $\eta$ increases. This ties well with our finding elaborated earlier. In Fig. 3, panels (a) and (c) show that the correct Go decision rates of the proposed method (adjusted) exceed those of the CI and PAR methods in all scenarios for both C$_\text {max}$ and AUC data. On the contrary, in the other biologically inequivalent scenarios, the percentage of correct No-Go decisions ascends as $\eta$ increases, since a high value of $\eta$ makes declaring bioequivalence harder (see Fig. 3 (b) and (d)). Combining the results from panels (a) and (b), one may consider setting $\eta = 0.8$ in the proposed method (adjusted) because this yields a high chance of 90.36% to give a correct Go decision under Sscenario 5 (i.e., GMR = 1.0) for C$_\text {max}$, which drops to 81.9% by setting $\eta = 0.85$, as reported in Table 1 and Fig. 2. The correct No-Go decision rate of the proposed models reaches 100% in scenarios with GMR = 0.5 and 2.0, which is the same as the CI and PAR methods. In the scenarios with GMR = 0.7 and 1.43, looking across Fig. 3 (b) and (d), setting $\eta$ to surpass 0.7 for C$_\text {max}$ and 0.5 for AUC means that the 1 - $\alpha$ of the proposed method (adjusted) exceeds 90%. The proposed method thus works extremely well to render correct Go and No-Go decisions, with $\eta$ set to an appropriate value following this pragmatic approach. More simulations have been performed under a different configuration of the prior mixture weight, with the results reported in Fig. S3.

We now carry on to interpret the results from our expanded simulation study that generates pivotal data following a Go decision given to the pilot trial under Scenario 5 (GMR = 1.0). The CI method is implemented using the pivotal data only. This is in contrast to implementing the proposed method that enables potential incorporation of pilot data in the final analysis on the completion of the pivotal trial. Here, we have assumed total 24 subjects would be enrolled. Table 2 displays the probability of declaring bioequivalence following the pivotal trial. Four options for specifying *w* are considered for illustration, that is, prior ambivalence ($w = 0.5$), low prior confidence ($w = 0.2$), minimal prior confidence ($w = 0.1$) in the exchangeability assumption, and no borrowing from pilot data permitted ($w = 0$). In all cases, we set $\eta = 0.75$, and the criterion in the form of Equation (8) is applied for the formal bioequivalence assessment. As Table 2 reveals, the proposed method enhances the chance of declaring bioequivalence substantially by incorporating the pilot data, as compared to the conventional CI method. Even if no borrowing from pilot data is permitted, the proposed method has around a 16% higher chance for the declaration for C$_\text {max}$, when the pivotal trial would suggest the two formulations are just bioequivalent (i.e., the borderline Cases A and E). In Case C (with GMR = 1.0), the probability of bioequivalence for C$_\text {max}$ almost reached 100%, for any level of prior confidence in the exchangeability assumption, whilst the CI method yields only 81.64%. Impressive operating characteristics are also observed for Cases B (GMR = 0.9) and D (GMR = 1.1): examining the C$_\text {max}$ data, for example, the proposed models provides at least 82.93%, compared to the CI method with only around 47.47-51.84%.

**Table 2:** Comparison between the proposed method and the conventional CI method in terms of the probability (%) of declaring bioequivalence on the completion of a pivotal trial. Pilot data are simulated based on the scenarios of GMR = 1.0, while pivotal data are simulated under Cases A - E with GMR = 0.8, 0.9, 1.0, 1.1, 1.25, respectively

Comparator	$\eta$	(*w*, 1 - *w*)	Case A	Case B	Case C	Case D	Case E
			GMR = 0.8	GMR = 0.9	GMR = 1.0	GMR = 1.1	GMR = 1.25
CI method ($\alpha$ = 5%)							
C$_{\text {max}}$			4.94	47.47	81.64	51.84	5.18
AUC			5.13	80.23	99.83	85.40	5.23
Proposed method							
C$_\text {max}$	0.75	(0.5, 0.5)	56.44	94.78	99.74	95.93	54.08
AUC	0.75	(0.5, 0.5)	52.65	99.14	100	99.56	52.15
C$_\text {max}$	0.75	(0.2, 0.8)	47.40	92.77	99.57	94.35	46.04
AUC	0.75	(0.2, 0.8)	43.62	98.76	100	99.23	42.39
C$_\text {max}$	0.75	(0.1, 0.9)	41.39	91.02	99.47	93.29	41.68
AUC	0.75	(0.1, 0.9)	38.21	98.40	100	98.98	37.49
C$_\text {max}$	0.75	(0, 1.0)	25.39	82.93	98.47	86.51	26.42
AUC	0.75	(0, 1.0)	25.08	97.08	100	98.29	26.09

**Fig. 4:**
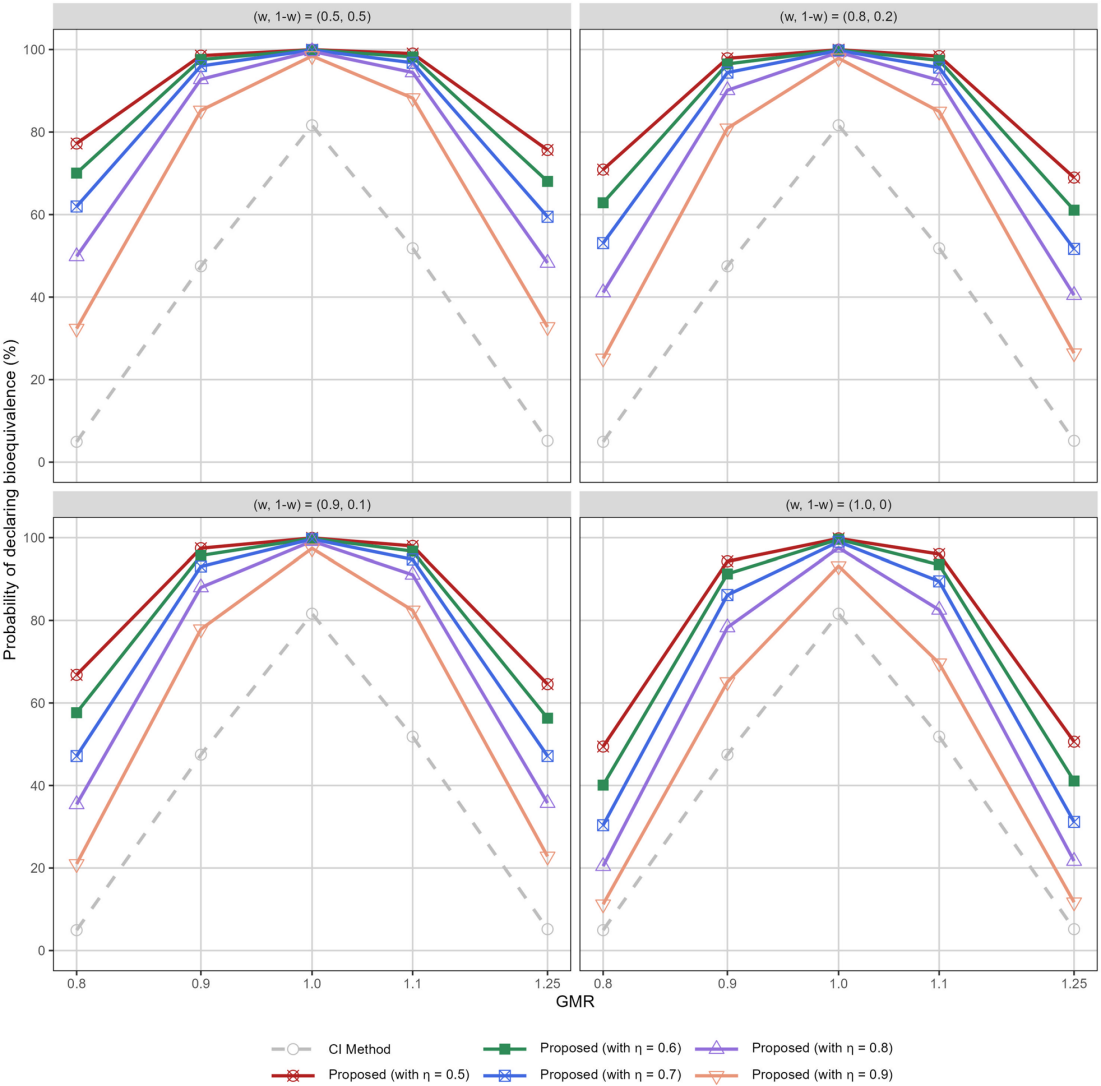
Comparison between the proposed method and conventional CI method in terms of the statistical power, i.e., probability of correctly declaring bioequivalence (%) on the completion of a pivotal trial, using C$_\text {max}$ data. Pilot data are simulated based on the scenarios of GMR = 1.0, while pivotal data are simulated under Cases A - E with GMR = 0.8, 0.9, 1.0, 1.1, 1.25, respectively

The effects of various configurations of $\eta$ and *w* on C$_\text {max}$ and AUC for bioequivalence evaluation can be found in Figs. 4 and S4. Across various values set for $\eta$, the probability of establishing average bioequivalence using the proposed method is higher than the conventional CI method in all truly equivalent scenarios. Finally, Table 3 reports the false positive rate of the analysis when the pivotal trial discloses inequivalence, in conflict to what the pilot data implies: as $\eta$ increases, the proposed method can maintain the probability of incorrectly declaring bioequivalence more effectively. This is applicable also for the boardline cases wherein the GMR = 0.8 or 1.25. For a strict control of false positive rate to be below 5%, practitioners may consider setting $\eta > 0.9$ as used in this illustration. On the other hand, when the prior mixture weight *w* deviates much from 1, it becomes more challenging to maintain the error rate due to the incorporation of overly optimistic pilot data suggesting bioequivalence. This suggests the proposed method can quickly discount inconsistent pilot data in the final analysis especially when $\eta$ and *w* can be set to values that are reasonably correct.

**Table 3:** Probability of incorrectly declaring bioequivalence (%) for pivotal trials analyzed using the proposed method. Pilot data are simulated based on scenario 5 wherein GMR = 1.0, while pivotal data are simulated under six additional cases that feature GMR =0.5, 0.7, 0.8, 1.25, 1.43, 2.0, respectively

	GMR	$\eta$	(*w*, 1 - *w*)			
			(0, 1.0)	(0.1, 0.9)	(0.2, 0.8)	(0.5, 0.5)
C$_\text {max}$	0.5	0.5	0	0	0	0
	0.5	0.6	0	0	0	0
	0.5	0.7	0	0	0	0
	0.5	0.8	0	0	0	0
	0.5	0.9	0	0	0	0
	0.7	0.5	3.61	9.11	11.68	17.19
	0.7	0.6	2.02	5.85	7.88	11.66
	0.7	0.7	1.15	3.44	4.85	7.45
	0.7	0.8	0.51	1.63	2.53	4.51
	0.7	0.9	0.12	0.61	0.86	1.58
	0.8	0.5	49.44	66.81	70.90	77.22
	0.8	0.6	40.09	57.64	62.86	70.05
	0.8	0.7	30.37	47.13	53.08	61.96
	0.8	0.8	20.45	35.42	41.11	49.87
	0.8	0.9	11.21	20.98	25.15	32.37
	1.25	0.5	50.62	64.55	68.97	75.65
	1.25	0.6	41.09	56.31	61.07	68.04
	1.25	0.7	31.17	47.14	51.68	59.47
	1.25	0.8	21.69	35.73	40.47	48.27
	1.25	0.9	11.70	22.81	26.40	32.76
	1.43	0.5	3.29	7.9	10.35	15.39
	1.43	0.6	1.83	4.87	6.49	10.30
	1.43	0.7	1.01	2.77	3.76	6.33
	1.43	0.8	0.41	1.46	1.94	3.40
	1.43	0.9	0.09	0.56	0.74	1.35
	2.0	0.5	0	0	0	0
	2.0	0.6	0	0	0	0
	2.0	0.7	0	0	0	0
	2.0	0.8	0	0	0	0
	2.0	0.9	0	0	0	0
AUC	0.5	0.5	0	0	0	0
	0.5	0.6	0	0	0	0
	0.5	0.7	0	0	0	0
	0.5	0.8	0	0	0	0
	0.5	0.9	0	0	0	0
	0.7	0.5	0.21	0.63	0.93	2.16
	0.7	0.6	0.09	0.37	0.55	1.10
	0.7	0.7	0.03	0.17	0.29	0.60
	0.7	0.8	0.03	0.06	0.12	0.27
	0.7	0.9	0	0.01	0.02	0.06
	0.8	0.5	49.83	62.58	65.77	72.77
	0.8	0.6	40.37	53.05	58.08	66.35
	0.8	0.7	30.01	43.48	49.04	57.85
	0.8	0.8	20.20	32.10	37.68	46.60
	0.8	0.9	10.58	19.49	23.76	31.35
	1.25	0.5	51.26	59.56	63.39	71.49
	1.25	0.6	41.06	51.05	55.58	64.56
	1.25	0.7	30.69	42.42	46.96	56.49
	1.25	0.8	21.43	32.49	37.64	47.38
	1.25	0.9	11.16	20.74	24.27	33.89
	1.43	0.5	0.16	0.41	0.73	1.55
	1.43	0.6	0.06	0.19	0.35	0.89
	1.43	0.7	0.03	0.10	0.17	0.41
	1.43	0.8	0.01	0.03	0.07	0.18
	1.43	0.9	0	0.02	0.02	0.06
	2.0	0.5	0	0	0	0
	2.0	0.6	0	0	0	0
	2.0	0.7	0	0	0	0
	2.0	0.8	0	0	0	0
	2.0	0.9	0	0	0	0

### Revisiting the Pantoprazole pilot trial

We now return to the motivating trial, and use our proposed Bayesian decision framework to analyse one simulated dataset of this study. We set $w = 0.9$ so that pilot data can be largely used to elicit a decision of Go or No-Go. The posterior means of $\theta _2$, with a 90$\%$ credible interval, are 0.1721 (-0.1131, 0.4507) and 0.0772 (-0.1332, 0.2776) obtained based on the C$_\text {max}$ and AUC measurements. See Table 4 for more summaries about the inference. Accordingly, $\exp \left( \theta _{2}\right)$ is 118.78% (89.31%, 156.94%) for C$_\text {max}$ and 108.03% (87.53%, 132.00%) for AUC, respectively. These contrast with the results of the conventional CI method, reported as 116.78% (with a 90% CI: 96.59% - 141.20%) for C$_\text {max}$ and 105.13% (93.10% - 118.72%) for AUC. The predictive probabilities of bioequivalence (thus with a Go decision assigned) are 68.1% and 90.0% using C$_\text {max}$ and AUC data, respectively. Furthermore, after the equivalence boundaries are modified by applying the scaling factor in the form of (7), the predictive probability of bioequivalence climbs to 83.1% for C$_\text {max}$ measurements. Note that the within-subject variance of C$_\text {max}$ is higher than AUC (i.e., 23.7% versus 12.2%), which justifies the wider confidence interval yielded by the CI method for the latter. Be that as it may, the degree of variance is acceptable in practice as it is still below the regulatory guideline’s threshold of high within-subject variability of 30% [25]. In this retrospective analysis, the proposed robust Bayesian model results in a higher chance to recommend a subsequent pivotal trial be conducted, unlike the conventional CI method that suffers severely from the data sparsity problem.

**Table 4:** Summary statistics of the predictive distribution of and the prediction about probability of declaring bioequivalence in pivotal trial, given the Pantoprazole tablet pilot trial example

Comparator	Mean	Standard deviation	Percentile		
			5.0%	50%	95.0%
C$_{\text {max}}$					
$\theta _2$	0.1721	3.0455	-0.1131	0.1543	0.4507
*exp (*$\theta _2$*)*	1.1878	21.0205	0.8931	1.1668	1.5694
pred.prob.be^1^	0.681	0.466	0	1.000	1.000
pred.prob.be^2^	0.831	0.375	0	1.000	1.000
AUC					
$\theta _2$	0.0772	3.0454	-0.1332	0.0510	0.2776
*exp (*$\theta _2$*)*	1.0803	21.0184	0.8753	1.0523	1.3200
pred.prob.be^1^	0.900	0.299	0	1.000	1.000
pred.prob.be^2^	0.905	0.293	0	1.000	1.000

^1^ Pred.prob.be $^1$ is the probability of making a Go decision to implement a pivotal trial, based on the unadjusted method, i.e., the proposed robust Bayesian hierarchical model with $\gamma$ = 1

^2^ Pred.prob.be $^2$ is the probability of making a Go decision to implement a pivotal trial, based on the proposed robust Bayesian hierarchical model with $\gamma$ defined in Eq. (7)

## Discussion

Bayesian statistics offers an attractive alternative to classical approaches for prediction and estimation. It has also been widely recognised as advantageous in using relevant information from historical studies for more informed analysis within a solid decision-theoretical framework. In this paper, we have established a robust Bayesian hierarchical model that accommodates the respective linear mixed-effects models fitted to the pilot and pivotal trials for bioequivalence assessment. Moreover, our novel Bayesian decision criteria are shown to be effective in both graduating a pilot study that suggests genuine bioequivalence with a Go decision and leveraging consistent, as well as discounting inconsistent, pilot data in the final analysis of the pivotal trial. We recommend including a non-exchangeability distribution to relate the study-specific direct treatment effects for robust inferences. Specification of the prior mixture weight, *w*, reflects skepticism about the plausibility of an exchangeability assumption, which may be set to a value close to 1 for obtaining a Go/No-Go decision after the pilot trial, but a lower value in the final analysis. The proposed Bayesian decision framework has novelty in the inclusion of a scaling factor, $\gamma$, which is defined based on the study sample sizes and intra-subject variations. This particularly benefits the inference about whether a pivotal trial should be undertaken.

Bayesian meta-analytic approaches have been considered to borrow historical data in phase I [23, 24] and phase II [21, 26] clinical trials. As far as we are aware, this paper represents a very first proposal to using pilot data in the pivotal trial with a crossover design to evaluate bioequivalence. Our methodology can potentially replace the TOST procedure, which is constrained by the limited sample sizes [10], in the inference of pilot trial data alone, or of both pilot and pivotal trial data. Revisiting the Pantoprazole pilot trial, the proposed Bayesian method would have led to a Go decision whilst a No-Go decision was made based on the TOST procedure. The simulation results have also affirmed the superiority of our model as compared to the traditional methods and one modified version, the PAR method. Surprisingly, no simulation study was performed when the PAR method was proposed [14]. The simulation study in our paper thus also fills this gap in the literature and further guides the specification of $\eta$ for desired trial operating characteristics.

We acknowledge that the TOST method, when implemented with a higher significance level, can yield desirable operating characteristics that are comparable to the proposed Bayesian model. However, determining the significance level to ensure the performance poses a challenging decision. This approach can be further limited under small sample sizes that yield low estimation accuracy. In contrast, the proposed Bayesian approach provides the ability to elicit a Go/No-Go decision as well as to analyze data from both trials. The specification of key parameters, such as *w* and $\eta$, is more naturally aligned with the intuition. Our simulation study suggests that the proposed methodology is moderately robust to data inconsistency between pilot and pivotal trials. When the chosen values deviate too far from the truth, it may experience difficulty in maintaining the false positive rate in the final analysis. To enable more effective down weighting of inconsistent pilot data, one may consider transforming the pivotal trial in a two- or multi-stage manner. More specifically, investigators may specify *w* based on the best guess to initiate the pivotal trial, but reestimate the value at interim analyses for satisfactory operating characteristics in the final analysis [24].

The sample size of 12 subjects in a pilot trial for bioequivalence assessment has been regarded as the rule-of-thumb in practice [27]. Nevertheless, different countries have different sample size requirements. For instance, the sample size in the United States is 12-36 cases [8], but in Europe it is at least 12 [3]. In our simulation study the pivotal trial has a sample size of 24, which is in line with Chinese guidelines for bioavailability and bioequivalence studies [28]. In this work, we have defined the scaling factor, $\gamma$, through the respective sample sizes in the pilot and pivotal trials. One area that deserves further research is sample size determination for a pivotal trial that uses available pilot data. On the other hand, the pivotal trial has been supposed to have a fixed-sample design. Further efficiency could be leveraged by considering adaptive designs that enables, e.g., early stopping and/or sample size re-estimation, at the interim. This is also an area where our future research looks towards.

## Conclusion

In conclusion, we have proposed a robust Bayesian meta-analytic approach that facilitates the integration of pilot bioequivalence data. The proposed Bayesian methodology is novel and effective in different stages of bioequivalence assessment. It can greatly enhance the decision-making process in bioequivalence trials at various stages, and would be useful to the pharmaceutical industry and regulators.

### Supplementary Information

**Additional file 1.**

## Data Availability

All data and programming code during the current study are available at the GitHub repository: https://github.com/yddrz/BE-Bayesian-approach-to-pilot-pivotal-trials.

## Abbreviations

AUC: Area under the plasma concentration-time curve
CI: Confidence interval
GMR: Geometric mean ratios
Cmax: Maximum concentration
PAR: Pilot Acceptance Range
TOST: Two one-sided test
