# Supplementary material for: A Bayesian approach to pilot-pivotal trials for bioequivalence assessment
Source: BMC Med Res Methodol. 2023 Dec 19;23:301. doi: 10.1186/s12874-023-02120-2 (PMC10729540; doi:10.1186/s12874-023-02120-2)
Supplement: Supplementary file 1 — Additional file 1. [file 12874_2023_2120_MOESM1_ESM.zip › Supplement Table S1.docx]

Supplement Table S1. Treatment effects and variability in Pantoprazole tablet pilot trial, based on an ANOVA analysis of C_max_ and AUC data.

| PK parameters | ***n*** | ***μ*_T_** | ***μ*_R_** | ***φ*** | ***ρ*** | $\boldsymbol{\sigma}_{\boldsymbol{s}}^{\boldsymbol{2}}$ | $\boldsymbol{\sigma}_{\boldsymbol{w}}^{\boldsymbol{2}}$ |
| --- | --- | --- | --- | --- | --- | --- | --- |
| C_max_ | 12 | 8.349 | 8.187 | 0.016 | 0.025 | 0.088 | 0.237 |
| AUC | 12 | 9.231 | 9.163 | 0.048 | 0.026 | 0.101 | 0.122 |
