# Supplementary material for: A Bayesian approach to pilot-pivotal trials for bioequivalence assessment
Source: BMC Med Res Methodol. 2023 Dec 19;23:301. doi: 10.1186/s12874-023-02120-2 (PMC10729540; doi:10.1186/s12874-023-02120-2)
Supplement: Supplementary file 1 — Additional file 1. [file 12874_2023_2120_MOESM1_ESM.zip › Supplement Table S2.docx]

Supplement Table S2. Simulation setup of the true value for *μ*_T_ to form different scenarios of the Geometric Mean Ratio (GMR). All scenarios stipulate the true value for *μ*_R_ = 8.187 for C_max_ and 9.163 for AUC.

| PK parameters | Sc. 1  GMR = 0.5 | Sc. 2  GMR = 0.7 | Sc. 3  GMR = 0.8 | Sc. 4  GMR = 0.9 | Sc. 5  GMR = 1.0 | Sc. 6  GMR = 1.1 | Sc. 7  GMR = 1.25 | Sc. 8  GMR = 1.43 | Sc. 9  GMR = 2.0 |
| --- | --- | --- | --- | --- | --- | --- | --- | --- | --- |
| C_max_ | 7.494 | 7.830 | 7.964 | 8.082 | 8.187 | 8.282 | 8.410 | 8.545 | 8.880 |
| AUC | 8.470 | 8.806 | 8.940 | 9.058 | 9.163 | 9.258 | 9.386 | 9.521 | 9.856 |
