# Supplementary material for: A Bayesian approach to pilot-pivotal trials for bioequivalence assessment
Source: BMC Med Res Methodol. 2023 Dec 19;23:301. doi: 10.1186/s12874-023-02120-2 (PMC10729540; doi:10.1186/s12874-023-02120-2)
Supplement: Supplementary file 1 — Additional file 1. [file 12874_2023_2120_MOESM1_ESM.zip › Supplementary Material_R3.pdf]

Existing approaches to analysing pilot data

The TOST procedure (α = 0.05, 0.1 or 0.2)

The TOST procedure accepts the test drug for a subsequent pivotal bioequivalence trial if and only if the (1-2α)×100% confidence interval for the geometric mean ratio of the AUC and/or C_{max} falls within an acceptance range, typically 80%-125% on a log-transformed scale. The (1-2α)×100% confidence interval for the direct treatment effect, denoted by $\delta = \mu_T - \mu_R$, is bounded by d_1 and d_2 where

$$d_1 = \delta - t_{(\alpha,\ n-2)}\hat{\sigma}_D\sqrt{m_1^{-1} + m_2^{-1}} \quad \text{and} \quad d_2 = \delta + t_{(\alpha,\ n-2)}\hat{\sigma}_D\sqrt{m_1^{-1} + m_2^{-1}} \tag{9}$$

with m_j being the number of subjects for sequence j = 1, 2 for the pilot trial, and $\hat{\sigma}_D$ the estimated variance of the within-subject differences. On the log-scale, the criterion to conclude bioequivalence is

$$\log(0.8) < d_1 < d_2 < \log(1.25) \tag{10}$$

The pilot acceptance range (PAR) method

The PAR method accepts the test drug for a subsequent pivotal trial if and only if the 90% confidence interval for the geometrical mean ratio of the AUC and/or C_{max} in the pilot trial falls within an adjusted interval. On the log-scale, the PAR method concludes bioequivalence if

$$\log(0.8\lambda^{-1}) < d_1 < d_2 < \log(1.25\lambda) \tag{11}$$

$$\lambda = \exp\left(\left[t_{(\alpha,\ n-2)}\sqrt{m_1^{-1} + m_2^{-1}} - t_{(\alpha,\ n-2)}\sqrt{n_1^{-1} + n_2^{-1}}\right]\hat{\sigma}_D\right) \tag{12}$$

with m_j being the number of subjects for sequence j = 1, 2 for the pilot trial and n_j for the pivotal trial, and $\hat{\sigma}_D$ the estimated variance of the within-subject differences.
