# Supplementary figures and images for: A Bayesian approach to pilot-pivotal trials for bioequivalence assessment
Source: BMC Med Res Methodol. 2023 Dec 19;23:301. doi: 10.1186/s12874-023-02120-2 (PMC10729540; doi:10.1186/s12874-023-02120-2)

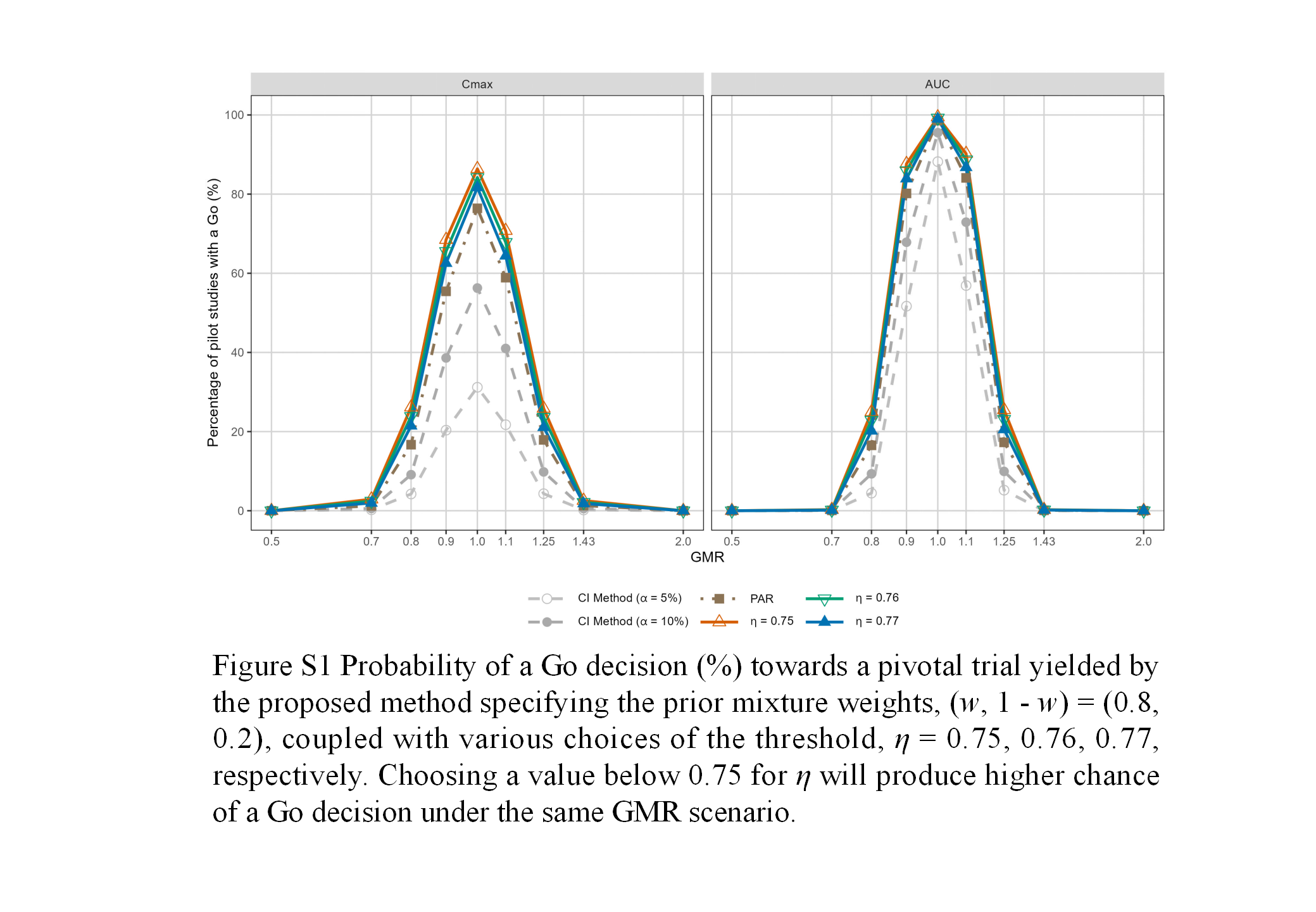

Supplement: Supplementary file 1 — Additional file 1. [file 12874_2023_2120_MOESM1_ESM.zip › Figure S1.tif]

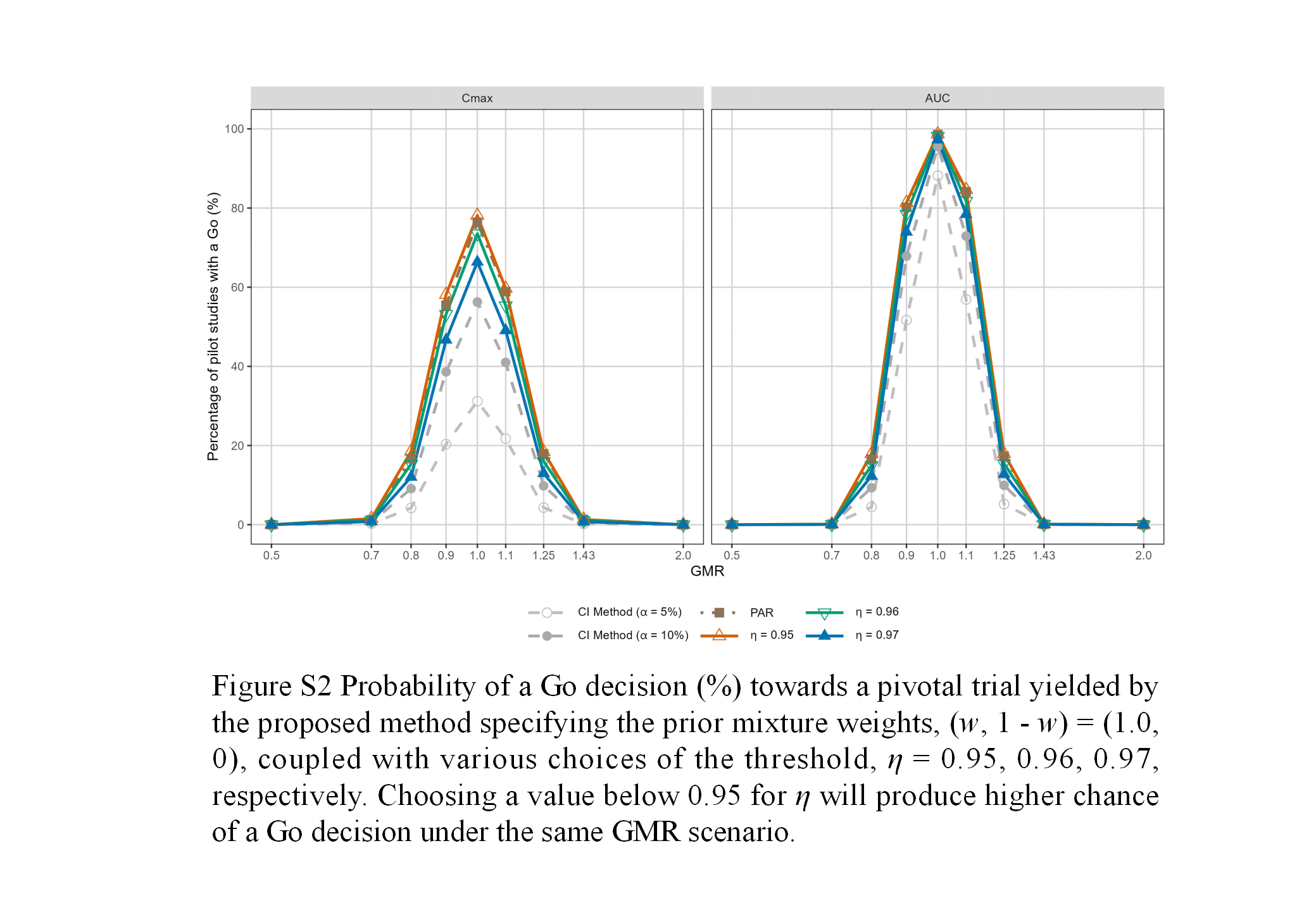

Supplement: Supplementary file 1 — Additional file 1. [file 12874_2023_2120_MOESM1_ESM.zip › Figure S2.tif]

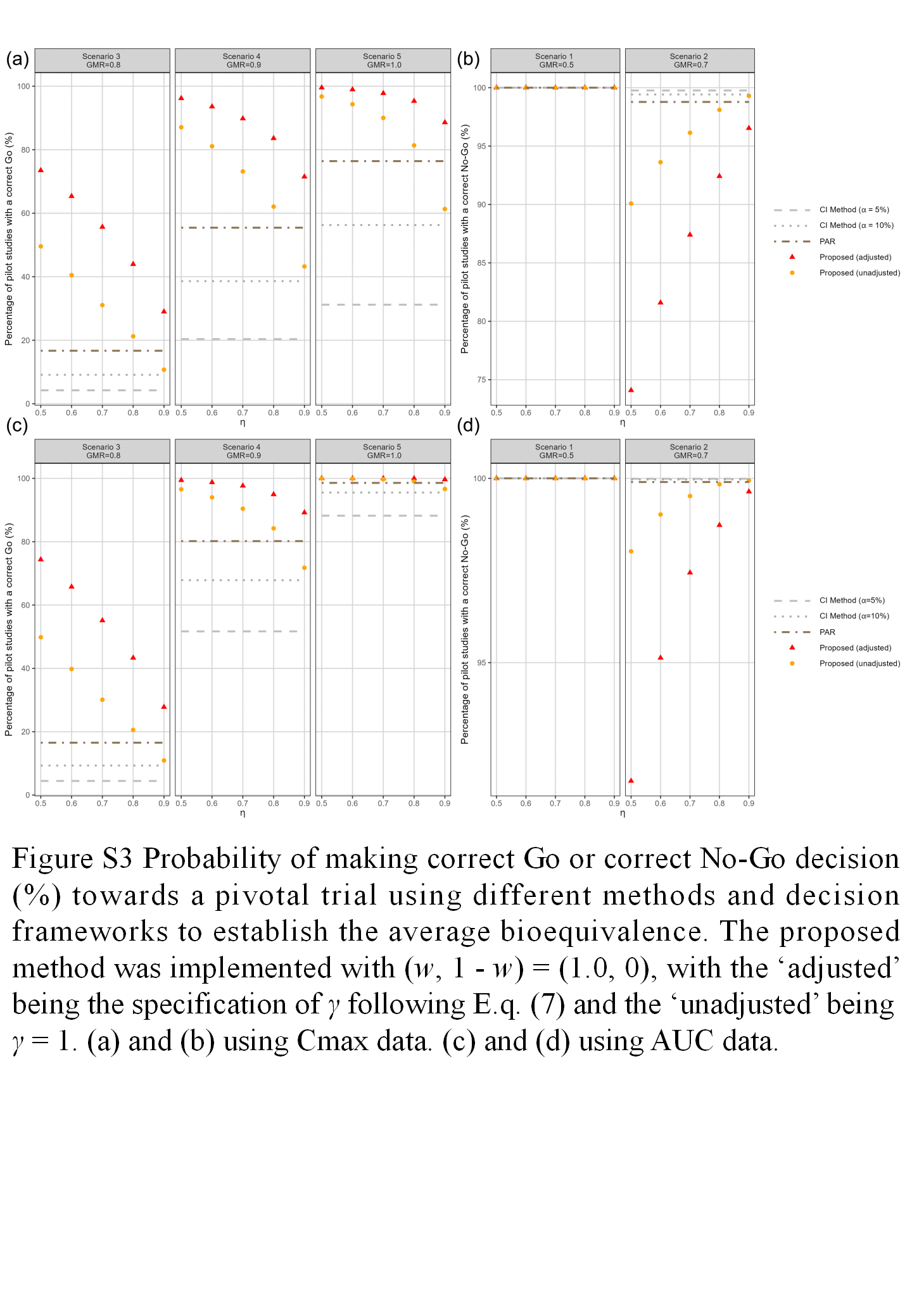

Supplement: Supplementary file 1 — Additional file 1. [file 12874_2023_2120_MOESM1_ESM.zip › Figure S3.tif]

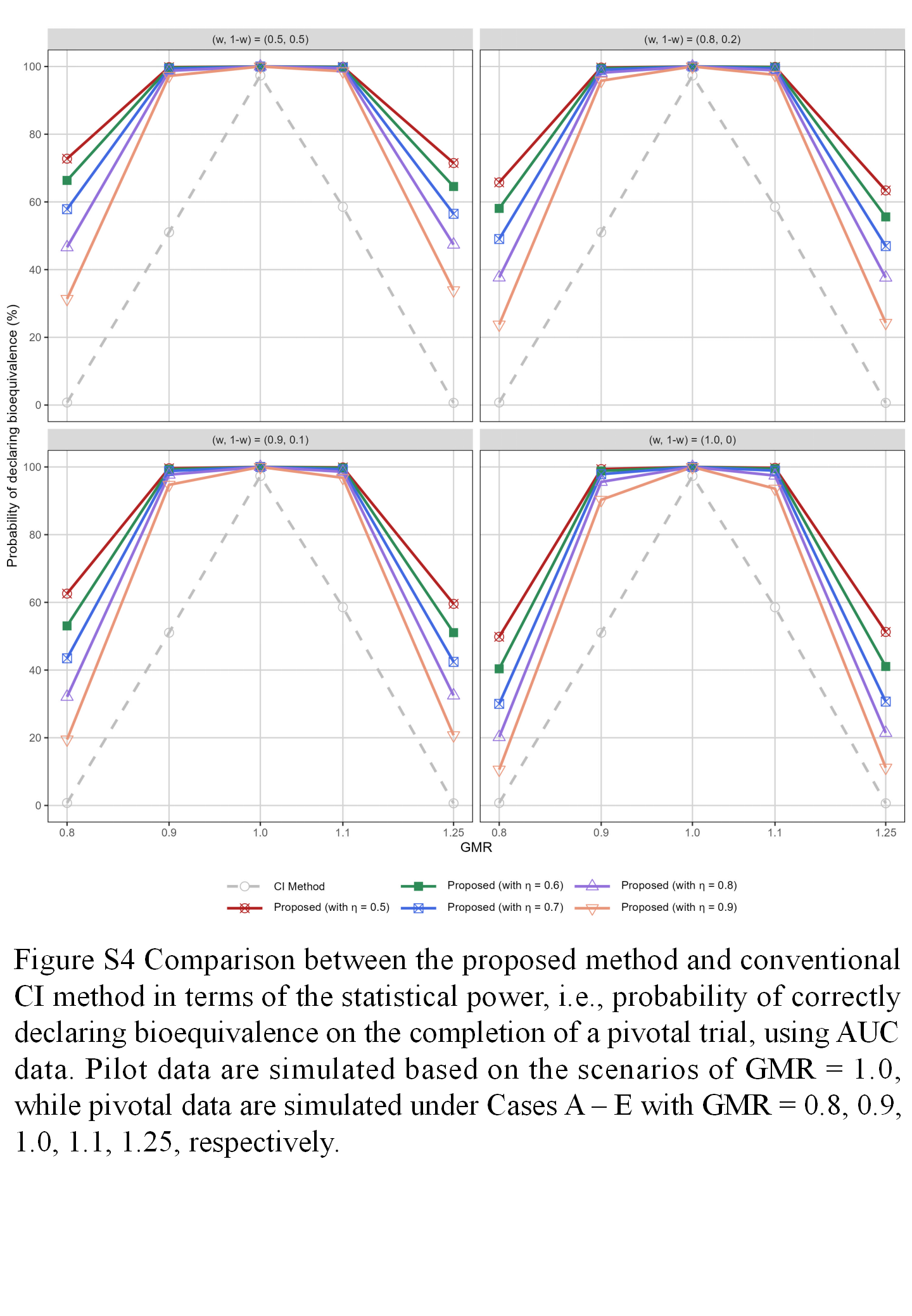

Supplement: Supplementary file 1 — Additional file 1. [file 12874_2023_2120_MOESM1_ESM.zip › Figure S4.tif]
